# Tongue carcinoma in an adult Down's syndrome patient: a case report

**DOI:** 10.1186/1477-7819-7-26

**Published:** 2009-03-04

**Authors:** Fadi S Farhat, Fady Geara, Mohamed Natout, Jamal Serhal, Walid Daya

**Affiliations:** 1 Hammoud Hospital University Medical Center Saida, Lebanon; 2 Mount Lebanon Hospital, Beirut, Lebanon; 3 American University Hospital, Beirut, Lebanon

## Abstract

**Background:**

Cancer of the oral cavity is rare and unusual in Down's syndrome patient. The over all risk is similar to that in adult population.

**Case presentation:**

This case report describes a 27 years old male with Down's syndrome, non-smoker, who developed a poorly differentiated squamous cell carcinoma of the tongue. The patient underwent a hemiglossectomy without neck dissection followed by a postoperative locoregional radiation therapy to a total tumor-bed dose of 56 Gy and 45 Gy to the neck. Three months later, the patient presented with local tongue recurrence and was treated by Docetaxel and Carboplatin chemotherapy with no significant response. The patient died one month later, 9 months after his initial diagnosis.

**Conclusion:**

To our knowledge, this is the first case of tongue carcinoma arising in a patient with Down's syndrome. This unique case might not be sufficient to make a significant conclusion on the prognosis and survival of these patients but will increase the awareness about this possibility and will help in the appropriate management of Down's syndrome patients.

## Background

The overall risk of cancer in individuals with Down's syndrome (DS) is similar to the normal population [1]. However, the distribution of tumor types in DS is very unusual, with leukemia constituting 60% of all cancers especially in children [2]. A recently published paper showed that having three copies of chromosome 21 reduces the incidence of solid tumors in people with Down's syndrome [3]. We report here an unusual case of tongue cancer in a young adult patient with DS in light of the available literature on solid tumors in DS patients.

## Case presentation

**Figure 1:**
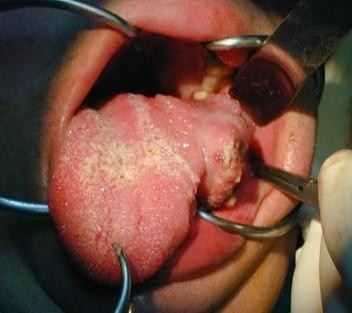
**Tumor in the left lateral oral tongue at presentation**.

**Figure 2:**
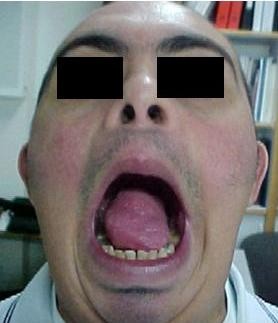
**No evidence of disease after surgery and radiotherapy**.

**Figure 3:**
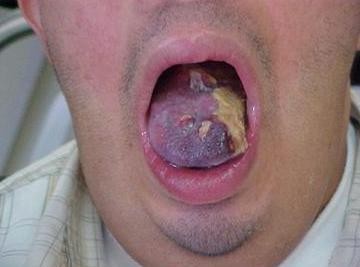
**Large tumor recurrence 3 months after postoperative radiotherapy**.

A 27 years old male with DS, non smoker and with no past medical history, presented to the otolaryngologist for hypersalivation and ulcer of his tongue. Clinical examination showed a mass of the lateral left aspect of the tongue measuring 4 × 3 cm (Figure 1) with no palpable neck lymph nodes. A biopsy was taken revealing a poorly differentiated squamous cell carcinoma. Chest X-ray, routine blood counts and chemistry were within normal range. The disease was staged T2N0M0. Two days later, the patient underwent a hemiglossectomy without neck dissection. Pathology revealed an irregular ulcerated lesion on the lateral surface of the tongue measuring 3.5 cm in maximal dimension compatible with squamous cell carcinoma infiltrating the tongue musculature with negative surgical margins. Postoperative loco-regional radiation therapy was delivered to a total tumor-bed dose of 56 Gy, and 46 Gy to the neck and there was no evidence of disease after surgery and radiotherapy (Figure 2). Three months post-radiation therapy and 7 months from the diagnosis, the patient presented with local recurrence that rapidly extended to the base of the tongue (Figure 3) and the diagnosis was confirmed by biopsy. The patient was offered radical salvage surgery that was declined by the patient and his family. The patient received 2 cycles of weekly Docetaxel (30 mg/m^2^) and weekly Carboplatin (area under the curve 4). Treatment was well tolerated with no nausea or neutropenia. However, evaluation at 6 weeks showed disease progression. Further chemotherapy was refused by the patient and his family. Supportive care was then initiated, along with a tracheostomy and a gastrostomy performed 2 months after the diagnosis of recurrence. The patient died one month later, 9 months after the initial diagnosis.

## Discussion

Down's syndrome or trisomy 21 is characterized by a recognizable phenotype. While people with DS have a high chance of developing childhood leukemia, a new study shows they have only half the normal lifetime risk of getting other kinds of cancer [3]. In a study of 2421 children with DS in Massachusetts, no cases of solid tumors were observed [4]. In another study report from the British Registry of Childhood Tumors, there were only seven patients with DS among 11000 cases of solid tumors [5]. In a European review of 6724 cases of children with neuroblastoma, none of the children had DS (while more than five would be expected in an age matched population) [6]. This was also the case in a similar study on 5854 children with Wilms' tumour [7].

This low risk of carcinomas in DS patients could be due to particular environmental exposure patterns, length of life of patients with DS, or possibly directly related to an inherent genetic effect. Indeed, several tumour suppressor genes have been identified on chromosome 21 [8] and a dosage effect of these genes on the extra copy of this chromosome could potentially exist. In addition, there is an increased susceptibility to apoptosis in cells derived from DS patients and this may result in higher rates of cell death rather than malignant transformation after major cell injury or exposure to mutagens [9]. Because the risk of breast cancer is almost inexistent in this population, women with DS have a lower risk of cancer than men. There is also a lower risk of esophageal and pulmonary cancer, which could be the result of limited exposure to occupational carcinogens, alcohol, and tobacco. Less sun exposure of the skin may also contribute to a decreased risk of skin cancer [9].

Cancer of the oral cavity is almost inexistent (or not reported) in individuals with DS. To our knowledge, no case(s) of squamous cell carcinoma of the tongue in DS patients were reported in the literature to-date. In our case report, the patient underwent a hemiglossectomy without neck dissection. A postoperative loco-regional radiation therapy was delivered to the neck to a total tumor-bed dose of 56 Gy, and 46 Gy. Radiation doses were slightly reduced from standard doses by 7% due to the underlying genetic disease and the potential risk of severe normal tissue reactions. Dose-fractionation range has been found to represent an adequate adjuvant postoperative dose for completely resected head and neck cancers [10]. Unfortunately, tumor recurrence developed rapidly after surgery. The reason for recurrence might be that patients with DS have a small mouth and a large tongue, thus access for performing partial glossectomy would have been difficult especially in the case of our patient who had a large tumor (3.5 cm on pathology). However, the pathology results reported the margins as negative and the physicians considered the margins as free of tumor and no new resection could be suggested at this time. Normally, T2N0 oral cavity tumors resected with negative margins do not require postoperative radiotherapy but our patient had two adverse clinicopathological features (the young age and the oral cavity tumor) necessitating post-operative radiotherapy.

Some reports indicate a lower survival rate for young patients (< 35 years) with oral cavity cancers with no DS compared to older patients [11], however these findings are inconsistent [12]. Data on recurrence patterns of oral cavity or other head and neck cancers in DS patients are unknown. DS patients, who are treated aggressively for myeloid leukemia, typically show better survival rates compared to patients without DS [9]. The outcome of our patient was markedly poor compared to what is observed in non-DS patients as he suffered rapid disease recurrence and deterioration in his general condition.

## Conclusion

This case illustrates an unusual tongue cancer in a young adult patient with DS. The patient suffered rapid recurrence after conventional therapy, which suggests that this rare type of solid tumors in DS patients may take an aggressive course. This unique case might not be sufficient to make a significant conclusion on the prognosis and survival of these patients but this report illustrates the unusual presentation of the disease and will shed a light on appropriate management of such patients.

## Competing interests

The authors declare that they have no competing interests.

## Consent

Written informed consent was obtained from the patient' parent for publication of this case report and any accompanying images. A copy of the written consent is available for review by the Editor-in-Chief of this journal.

## Authors' contributions

FF is the oncologist and principal investigator, who prepared, organized, wrote, and edited all aspects of the manuscript. FG was the radiotherapist on the case and helped in preparing the manuscript. MN and JS were the otolaryngologists who performed the first and second surgery on the patient. WD was the pathologist on the case, and helped with pathological sections in the manuscript. All authors have read and approved the final version of the manuscript.
